# On the Inside

**DOI:** 10.1093/plphys/kiab265

**Published:** 2021-07-29

**Authors:** Peter V Minorsky

**Affiliations:** School of Health and Natural Sciences, Mercy College, Dobbs Ferry, New York, USA

## Polygalacturonase affects grapevine stem hydraulics

Pierce’s Disease is a deadly disease of grapevines (*Vitis vinifera*) caused by *Xylella fastidiosa*, a bacterium that is spread by certain species of xylem feeding leafhoppers. During its colonization of the xylem network, *Xylella* forms a biofilm within the intervessel pit fields and secretes cell wall-digesting enzymes (e.g., polygalacturonase) to breach the pit membranes, thereby facilitating migration of the bacteria to adjacent vessels. While pit membranes impose a resistance to water flow, they also are believed to prevent the spread of air bubbles that may arise during extended periods of water deficit. As such, plant vascular systems may be compromised during *Xylella* infection by either drought-induced embolism, clogging by the vascular pathogens themselves, or both, ultimately leading to hydraulic dysfunction. On the other hand, with the pit membranes pores enlarged, the effects on water transport should lead to both higher xylem hydraulic conductivity and lowered resistance to axial flow. As a further complication, the presence of *Xylella* in the xylem may trigger ethylene production and a subsequent production of tyloses. To eliminate the effects of both the presence of *Xylella* in the xylem and the plant response to *Xylella* (i.e., the production of both ethylene and tyloses), Fanton and Broderson (pp. 1919–1931) perfused the xylem of grapevines with polygalacturonase alone, an enzyme known to be produced by the bacterial biofilm. Six grapevine genotypes with varying levels of resistance to Pierce’s Disease were studied, with the expectation that pit membrane resistance to degradation by polygalacturonase may play a role in resistance to this disease. The authors report that pit membrane damage did occur in stems perfused with polygalacturonase alone. Although the damaged pit membrane area was small, membrane digestion led to significant changes in air-seeding thresholds and a universal reduction in stem hydraulic conductivity, suggesting that the development of tyloses and the presence of the bacteria themselves may not be the only contributing factors to reduced hydraulic conductivity in infected grapevines.

## Regulation of the floral transition in *Dendrobium* orchids

Most orchid genera, including the commercially popular genus *Dendrobium*, experience a long vegetative phase that can last more than a year. This lengthy vegetative growth phase is a major obstacle for orchid breeding, and is poorly understood. By means of gene transformation and an in vitro tissue culture system, Li et al. (pp. 2021–2036) have successfully generated transgenic *Dendrobium* orchids, in which Dendrobium Orchid TERMINAL FLOWER1 (DOTFL1), an ortholog of TFL1 in Arabidopsis (*Arabidopsis thaliana*), is either overexpressed or downregulated. TFL1 is a phosphatidylethanolamine-binding protein, and its orthologs in other species are known to function in regulating diverse developmental processes, including seed development, flowering time, and inflorescence development. In some species, TFL1 suppresses floral transitioning, and this appears to be the case in *Dendrobium* as well. The authors report that the overexpression of *DOTFL1* in *Dendrobium* delays flowering and pseudobulb formation and produces defective inflorescence meristems and flowers with vegetative traits. In contrast, the knockdown of *DOTFL1* accelerates flowering and perturbs the maintenance of the inflorescence meristem. These findings suggest that DOTFL1 promotes vegetative growth in *Dendrobium* and has a previously unknown role in pseudobulb formation in the Orchidaceae.

## Insights into the tradeoff between plant growth and stress responses

High-temperature stress and pathogen attack are frequently encountered by plants growing in tropical or subtropical climates and can lead to severe retardation in growth and development, sometimes even death. To maximize fitness, such plants need to prioritize the allocation of their limited resources between growth and stress responses. The resulting trade-off is thought to be regulated by crosstalk between signaling pathways. The NAC family of transcription factors have been implicated in the regulation of plant responses to stress. Cai et al. (pp. 2169–2189) now describe the mechanism underlying trade-offs between growth, immunity, and thermotolerance in pepper (*Capsicum annuum*) with special emphasis on NAC. NAC-type transcription factor CaNAC2c was induced both by thermal stress and by infection with *Ralstonia solanacearum*, a pathogenic bacterium. CaNAC2c-inhibited growth and promoted immunity against *Ralstonia* by activating jasmonate-mediated immunity and H_2_O_2_ accumulation. In a markedly different manner, CaNAC2c promoted thermal stress responses by activating Heat Shock Factor A5 (*CaHSFA5*) transcription and blocking H_2_O_2_ accumulation. In response to thermal stress, CaNAC2c-CaHSP70 interaction in the nucleus protected CaNAC2c from degradation and resulted in the activation of thermotolerance by increasing CaNAC2c binding and transcriptional activation of its target promoters. In contrast, in response to bacterial infection, CaNAC2c interacted with CaNAC029 in the nucleus and activated jasmonate-mediated immunity but was prevented from activating thermotolerance-related genes. Thus, CaNAC2c physically interacts with either CaHSP70 or CaNAC029 in a context-specific manner.

## Dynamics of hydraulic conductance during recovery from drought

Xylem cavitation, due to the formation of xylem embolisms, is thought to be the main factor limiting the hydraulic conductance of plant organs during acute episodes of drought. There are reports, however, that decreases in root hydraulic conductance often precede the formation of xylem embolisms. These pre-embolism declines in hydraulic conductance in response to mild drought stress have been attributed to a range of factors including, cortical lacunae formation involving cellular dehydration and collapse, suberization, and reduced aquaporin activity. The extents of these varied root responses, all of which lead to reduced hydraulic conductivity, have been found to increase with increasing water stress severity, suggesting perhaps that the severity of water stress may dictate the speed of root recovery responses following soil water replenishment. It has also been found that in response to intensifying soil water deficit in the soil, the root hydraulic conductance of some plant species decreases, triggering stomatal closure. However, it is unknown in such cases, how the dynamics of hydraulic conductance recovery following rewatering after drought might influence gas exchange. Bourbia et al. (pp. 1908–1918) have examined the decline and recovery of the whole root hydraulic conductance and canopy conductance during exposure to moderate water stress in two species with contrasting types of root systems: *Tanacetum cinerariifolium* (herbaceous Asteraceae) and *Callitris rhomboidea* (woody conifer). Optical dendrometers were used to record stem water potential at high temporal resolution and enabled noninvasive measurements of hydraulic conductance. During the early stages of water stress, there were parallel declines in both species in both hydraulic conductance and canopy conductance parameters. The recovery of both the hydraulic conductance and the canopy conductance after rewatering, however, differed between the two species. *Tanacetum cinerariifolium* recovered relatively quickly, while the recovery of *C. rhomboidea* was much slower. These findings suggest that the pronounced sensitivity of hydraulic conductance to drought is a common feature among different plant species, but recovery may vary between species.

## Molecular insights into dark-induced leaf senescence

A group of NAC domain-containing transcription factors, including ANAC017, have been shown to play an important role during retrograde signaling in plants. Knock-out mutants of *ANAC017* show strongly repressed mitochondrial retrograde signaling. Recent studies in Arabidopsis (*Arabidopsis thaliana*), however, have reported conflicting roles for ANAC017, and its closest paralog NAC DOMAIN CONTAINING PROTEIN 16 (ANAC016), in the process of leaf senescence. Unlike these previous studies, Broda et al. (pp. 2205–2221) have used a different experimental approach to determine the role of ANAC017 in plant senescence. By darkening only individual attached leaves, while keeping the rest of the plant under optimal conditions for plant growth, the authors were able to analyze dark-induced senescence, while maintaining systemic communication, a situation that mimics partial shadowing by a neighboring plant. By comparing the different *ANAC017* mutants that had been used in the previous conflicting studies, the authors provide clear evidence that the overexpression of *ANAC017* positively regulates leaf senescence and cell death. A time-resolved transcriptome analysis revealed that senescence-associated pathways such as autophagy are not constitutively activated in *ANAC017* overexpression lines but require a senescence-stimulus to trigger accelerated induction. *ANAC017* transcript and ANAC017-target genes are constitutively upregulated in *ANAC017* overexpression lines, whereas knockout mutants of *ANAC017* showed lowered senescence-induced induction of ANAC017 target genes during the late stages of dark-induced senescence.

## Increasing thiamin levels in plants via metabolic engineering

Thiamin (or thiamine) is a water-soluble B-vitamin (B1), which is required, in the form of thiamin pyrophosphate (TPP), as an essential cofactor in many crucial enzymatic steps in carbon metabolism, including glycolysis, the tricarboxylic acid cycle, nucleotide metabolism, and the synthesis of branched-chain amino acids. Humans, lacking the ability to synthesize this vitamin de novo, require adequate dietary supplies of B1 vitamins to ensure normal cell functioning. Unfortunately, thiamin deficiency is a persistent global health problem that can lead to neurodegenerative and cardiovascular pathologies. Increasing thiamin levels in plants via metabolic engineering may help to alleviate vitamin B1 malnutrition and thus improve global human health. These engineering strategies rely on comprehensive knowledge of plant thiamin metabolism and its regulation. With this goal in mind, Strobbe et al. (pp. 1832–1847) have examined multiple metabolic engineering strategies in the model plant *Arabidopsis thaliana*. This was achieved by the constitutive overexpression of three biosynthesis genes responsible for B1 synthesis, *HMP-P synthase*, *HET-P synthase*, and *HMP-P kinase/TMP pyrophosphorylase*, either separately or in combination. By monitoring the levels of thiamin, its phosphorylated entities, and its biosynthetic intermediates, the authors provide insight into the efficacy of these strategies in enhancing thiamin biosynthesis. A general conclusion reached by these studies is the necessity of balancing the pyrimidine and thiazole branches of thiamin biosynthesis. A second finding of importance is that the accumulation of certain metabolites appears to be tolerated in planta, whereas others are subject to stricter control mechanisms. These results are an important step toward acquiring a better understanding of the complex regulation of plant B1 biosynthesis and can serve as a guideline to develop novel B1 metabolic engineering strategies.

